# Nitrogen and Carbon Reallocation in Fungal Mycelia during Decomposition of Boreal Forest Litter

**DOI:** 10.1371/journal.pone.0092897

**Published:** 2014-03-20

**Authors:** Johanna B. Boberg, Roger D. Finlay, Jan Stenlid, Alf Ekblad, Björn D. Lindahl

**Affiliations:** 1 Uppsala BioCenter, Department of Forest Mycology & Plant Pathology, Swedish University of Agricultural Sciences, Uppsala, Sweden; 2 Örebro Isotope Laboratory, School of Science and Technology, Bilberg Building, Örebro University, Örebro, Sweden; University of Nebraska-Lincoln, United States

## Abstract

Boreal forests are characterized by spatially heterogeneous soils with low N availability. The decomposition of coniferous litter in these systems is primarily performed by basidiomycete fungi, which often form large mycelia with a well-developed capacity to reallocate resources spatially- an advantageous trait in heterogeneous environments. In axenic microcosm systems we tested whether fungi increase their biomass production by reallocating N between *Pinus sylvestris* (Scots pine) needles at different stages of decomposition. We estimated fungal biomass production by analysing the accumulation of the fungal cell wall compound chitin. Monospecific systems were compared with systems with interspecific interactions. We found that the fungi reallocated assimilated N and mycelial growth away from well-degraded litter towards fresh litter components. This redistribution was accompanied by reduced decomposition of older litter. Interconnection of substrates increased over-all fungal C use efficiency (*i.e.* the allocation of assimilated C to biomass rather than respiration), presumably by enabling fungal translocation of growth-limiting N to litter with higher C quality. Fungal connection between different substrates also restricted N-mineralization and production of dissolved organic N, suggesting that litter saprotrophs in boreal forest ecosystems primarily act to redistribute rather than release N. This spatial integration of different resource qualities was hindered by interspecific interactions, in which litters of contrasting quality were colonised by two different basidiomycete species. The experiments provide a detailed picture of how resource reallocation in two decomposer fungi leads to a more efficient utilisation of spatially separated resources under N-limitation. From an ecosystem point of view, such economic fungal behaviour could potentially contribute to organic matter accumulation in the litter layers of boreal forests.

## Introduction

In boreal forests, which cover a major part of the terrestrial northern hemisphere, saprotrophic fungi play an important ecological role as litter decomposers. The organic litter layers of boreal forests are characterized by a high degree of stratification, where C:N ratios decrease with increasing litter age and depth [1]. As litter components are used sequentially in order of decreasing accessibility [2], the usability of the residual litter as a resource for fungal growth (hereafter referred to as “quality”) also decreases. The filamentous growth form and the well-developed capacity of many litter fungi to translocate carbohydrates and nutrients within their mycelia enable efficient exploitation of such spatially separated substrates [3], [4]. Thus, fungal translocation may mediate interaction between interconnected substrates of different quality with subsequent implications for overall turnover of C and N in boreal forests.

In boreal forests, mineral nutrients, particularly N, are usually scarcely available and primary production is generally N limited [5]. Due to the low N content of coniferous litter, decomposition of recently shed litter also appears to be N limited [6], and low levels of exogenous N addition have been observed to increase colonization and decomposition by fungi in laboratory studies [7], [8], [9]. During the first years of decomposition, the N content of the litter frequently increases, not only in relative concentrations but also in absolute amounts [2], [10], [11], [12]. This increase has been attributed to fungal N import [13], [14], [15] in order to overcome N limitation of growth and activity in newly shed litter [16], [17].

In the same way as fungal translocation of N could support colonization of fresh, high C:N ratio litter, fungal C translocation could also enable more effective utilization of low C:N ratio substrates. In low C:N ratio substrates, N is conventionally assumed to be mineralized as a result of C-limitation of the decomposer microorganisms. N-containing organic compounds are then used as a C source rather than an N source, and excess NH_4_ is released to the environment. However, in microcosm studies, Boberg et al. [9] showed that litter decomposing fungi may be able to overcome local C limitation by reallocating C from fresh needle litter, preventing N-mineralization.

Not only the plant derived substrates, but also the fungal communities are spatially heterogeneous and display vertical zonation [1], [18]. Interspecific interactions, generally involving antagonism and combative behaviour, constantly take place in natural communities, as different individuals and species compete for space and resources [19], [20]. Interspecific interactions may restrict the potential for resource reallocation between substrates of different quality and thereby have major consequences for decomposition and nutrient mineralization.

Here we aim to test the following specific hypotheses; (i) that N would be reallocated from well decomposed needles to fresh litter, with concomitant increases in fungal growth and needle decomposition in the fresh litter, (ii) that C would be reallocated from fresh litter to well degraded litter, increasing fungal growth and needle decomposition as well as reducing N losses (N-mineralization), and (iii) that interactions between different fungal species would prevent C and N translocation between litter components.

**Figure 1:**
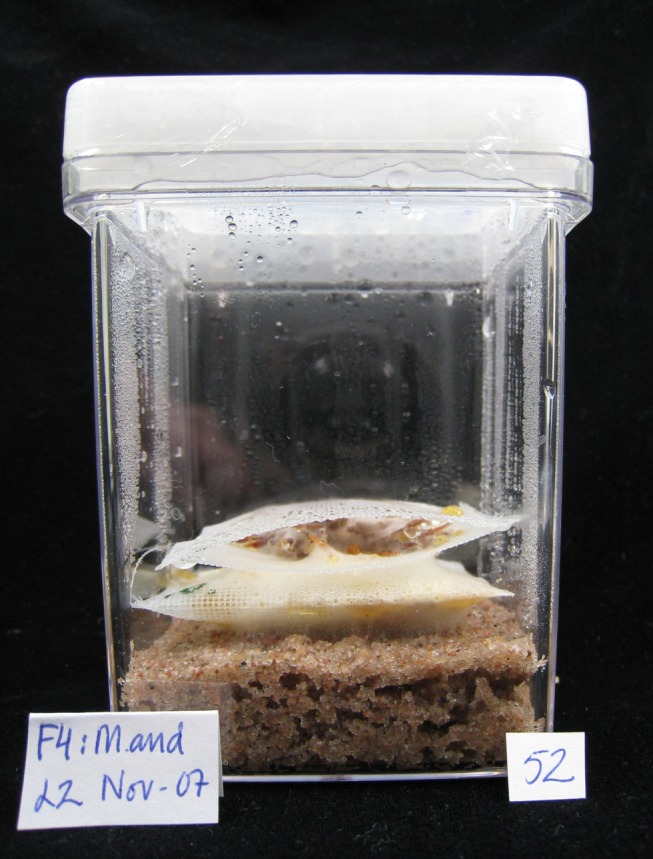
Axenic laboratory systems used in the study. Microcosm with *Pinus sylvestris* needles in litter bags inoculated with *Gymnopus androsaceus* (lower, ‘old litter’) and *Mycena epipterygia* (top, ‘new litter).

In order to test these hypotheses, we used a microcosm approach to obtain a detailed, integrated picture of resource dynamics and redistribution between natural substrates under controlled conditions. Axenic laboratory microcosms were prepared with Scots pine (*Pinus sylvestris*) needle litter inoculated with the litter decomposing basidiomycetes *Gymnopus androsaceus* or *Mycena epipterygia* (Fig. 1). Species belonging to these genera occur abundantly in decomposer communities in forest litter [1], [21], [22] and are efficient decomposers of both cellulose and lignin [23]. The systems were constructed so that portions of litter at different stages of decomposition were colonized by common fungal mycelia, allowing translocation of resources. Patterns of resource reallocation were compared between single-species systems and two-species systems involving antagonistic interactions between different fungal individuals. As ascomycetes in the order Helotiales are common components of litter colonising communities [1], interaction with a Helotialean species was also included.

## Material and Methods

### Fungal species, substrates and experimental design

Three species of litter fungi were used in the experiment; *Gymnopus androsaceus* (L.) J.L. Mata & R.H. Petersen (earlier *Marasmius androsaceus* (L.: Fr.) Fr.) isolate JB14, *Mycena epipterygia* (Pears.) Kühn., isolate JB13, and an unknown species within the order Helotiales (isolate BDI). The different species of fungi will hereafter be referred to as Gymnopus, Mycena and Helotiales, respectively. All isolates are deposited at the Department of Forest Mycology and Plant Pathology, SLU, Sweden. The strains and their decomposition capacity were previously described in Boberg *et al.* [23], and ITS sequences are deposited at NCBI (GU234007, GU234008 and GU393951).

Brown, abscised Scots pine needles were obtained from the yearly collection of needles at Jädraås experimental forest, central Sweden (60°49′N, 16°30′E) [24] with permission from the Swedish University of Agricultural Sciences. The needles were collected on sheets in plot Ih0, which consists of a 25-year-old pine stand. After collection, the needles were air dried and stored at −27°C. The needles contained 0.4% N, had a C/N-ratio of 135, and a lignin concentration of approximately 25% [25].

Microcosms were constructed, in which sand, litter and fungi were spatially organised to represent the vertical zonation of boreal forest litter layers (Fig. 1). In order to keep litter cohorts at different stages of decomposition separated, portions of 0.6 g dw of needles were placed in Polyester mesh bags (5×5 cm; 0.6 mm mesh, Sintab Produkt AB, Oxie, Sweden). A small bundle (Ø 1 cm) of glass wool was placed within each bag next to the needles. The pure fungal mycelium growing into the glass wool was used to determine ratios of chitin to fungal C and N. The filled litter bags were dried at 80°C for 72 h, weighed, and thereafter autoclaved at 121°C for 15 min. The sterilised needle-filled bags were placed on 85 g acid washed and autoclaved sand (Baskarpsand B55, Askania, Gothenburg, Sweden) in 77 mm×77 mm×97 mm plastic containers (Magenta corporation, Chicago, US; Fig. 1). The systems were inoculated by placing a pair of *Pinus sylvestris* needles, pre-inoculated on water agar, under the needle filled bag. Some systems were inoculated with Gymnopus (n = 47) and the rest with Mycena (n = 47). A subset of the systems was supplemented with 18 ml of 7 mM NH_4_Cl solution labelled with 1 atom% ^15^N excess of ^15^NH_4_Cl (Cambridge Isotope Laboratories, Andover, USA) added to the sand. To the remaining systems, 18 ml of 7 mM NaCl were added to ensure equal osmotic and Cl^−^ concentrations. The vessels were closed with lids, sealed with Parafilm and incubated at 20°C in darkness for 146 days. The vessels were ventilated every 30 days in a sterile environment.

**Figure 2:**
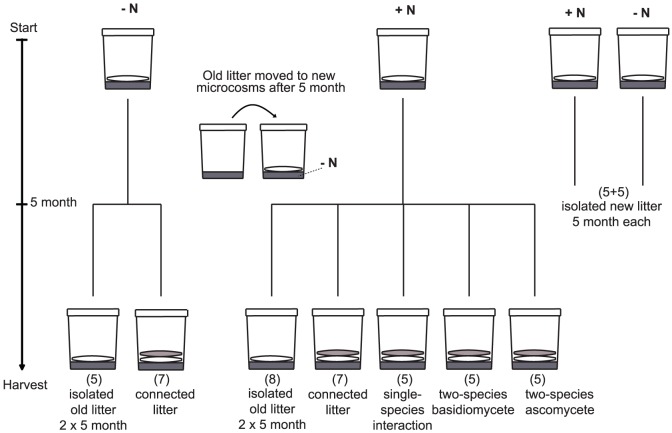
Experimental design. Illustration of the experimental design showing the time line and a description of the sequential treatments. At start, litter bags filled with *Pinus sylvestris* needles inoculated with either *Gymnopus androsaceus* or *Mycena epipterygia* were placed directly on sand receiving ^15^NH_4_Cl (+N) or NaCl (-N). After 5 months the old litter (white) was moved to new vessels with sand receiving NaCl and a second layer of new litter (grey), uninoculated (connected litter), inoculated with the same species (single-species interaction), inoculated with the other species, *G. androsaceus* or *M. epipterygia*, (two-species basidiomycete) or a Helotiales sp. (two-species ascomycete), was placed on top of the old litter. In some systems, the old litter was incubated without addition of a second litter layer (isolated systems). After an additional 5 months, the experiment was terminated. Control systems with litter bags were incubated in isolation for 5 month only, were also prepared. Number of replicates for each treatment is indicated in brackets.

After the first incubation period, the litter bags were moved to a new set of vessels prepared with 85 g of acid washed and autoclaved sand wetted with 18 ml of 7 mM NaCl solution. The new vessels were then treated as follows (Fig 2): For each of the four treatments (two fungi, with or without added N), some systems were supplemented with a second un-inoculated needle-filled litter bag, added on top of the first one (connected systems). Other systems received no second litter bag (isolated systems). In order to investigate effects of interspecific interactions, some systems (with ^15^N added during period one) were supplemented with a second needle bag that had been pre-inoculated (30 days on water agar) with either Gymnopus, Mycena or Helotiales. In addition, isolated systems were prepared for only one incubation period. All vessels were sealed and incubated at 20°C in darkness for another 151 days (second incubation period), after which they were harvested. The needle bags were carefully removed and washed in 10 ml of ultra-pure water (Milli-Q, Merck Millipore), in order to remove all exudates. The 10 ml of washing solution was poured into the sand and the boxes were stored at −27°C. The needle bags were dried at 80°C for 72 h, weighed, and ground in a ball mill.

### Analyses

Fungal biomass in the needles was determined by chitin analysis using a method described by Ekblad and Näsholm [26]. Fungal mycelium that had grown into the glass wool bundles (pooled within each treatment) was used to establish conversion factors between fungal chitin, C and N, which were subsequently used to calculate actual needle decomposition (after subtraction of accumulated fungal mass) and fungal C use efficiency. Both needles and glass wool bundles were ground in a ball mill and the contents of C, N and ^15^N were analysed on a CN analyzer (model EuroEA3024, Eurovector, Milan, Italy) coupled to an Isoprime isotope-ratio mass spectrometer (GV Instruments, Manchester, UK). The amount of mycelium attached to the mesh bags after removing the needles was estimated gravimetrically.

Losses of C and N to the sand were determined as total dissolved organic carbon (DOC) and nitrogen (DON), and N mineralization was estimated as NH_4_ produced in the sand solution. The sand from each vessel was extracted with 43–50 ml of ultra-pure water by shaking for 2 h. Sand solution was filtered through glass fibre filters (Type A/C Pall Life Sciences) and analysed for NH_4_ content on a flow injection analyser (FIAstar, Foss Tecator, Höganäs, Sweden). DOC and DON were determined using a CN analyser (Shimadzu TOC-VCPH coupled to a TNM-1, Japan). The ^15^N content of the DON pool was determined in pooled and evaporated samples of treatments receiving N addition.

### Calculations and statistical analysis

The chitin content of the needles was corrected for pre-experimental amounts and subsequently converted to fungal C using conversion factors, which were obtained by linear regression of data from glass wool bundles. Needle decomposition was then calculated as the sum of needle mass loss and fungal biomass production.

Total N in needles and mycelium was calculated as the sum of N in needles with associated mycelium and N in mycelium attached to the litter bags. Total ^15^N in needles and mycelium was calculated in the same way and converted to ^15^N excess by subtracting natural ^15^N abundances.

Fungal biomass is expressed as mycelial C and was calculated as the total amount of C in mycelium in needles (as estimated by chitin analysis) and on the litter bag (estimated gravimetrically assuming a C content of 50%). Decomposition, total N in needles and mycelium, and fungal biomass are all expressed per amount needles at start. C-use efficiency was calculated as the ratio of total mycelial C to total C decomposed for whole systems at harvest.

DOC and DON were expressed in relation to the amounts of needle C degraded during the second period, since the amounts of DOC and DON lost during the first incubation period were not determined. The contribution of needle N and fungal N to DON was calculated from the ^15^N content of the DON, un-colonized needles and fungal mycelium in the glass wool bundles, using a mixing model [27].

Data on fungal biomass, NH_4_, DOC, DON and C-use efficiency were log-transformed before statistical analysis, to meet the assumption of normality and homogeneity of variance. Effects of species, addition of N and litter bag connections, as well as single-species and two-species interactions, on decomposition, fungal biomass production, total N content, DOC, DON and NH_4_ production were tested using ANOVA. Effects of species, N addition and litter bag connections on C-use efficiency were also tested using ANOVA. In cases where significant explanatory variables in the ANOVA had more than two categories or when interaction terms were significant, Tukey's or Fisher's LSD post-hoc tests were used at the 0.05 significance level.

## Results

### N-reallocation, mycelial growth and litter decomposition

**Figure 3:**
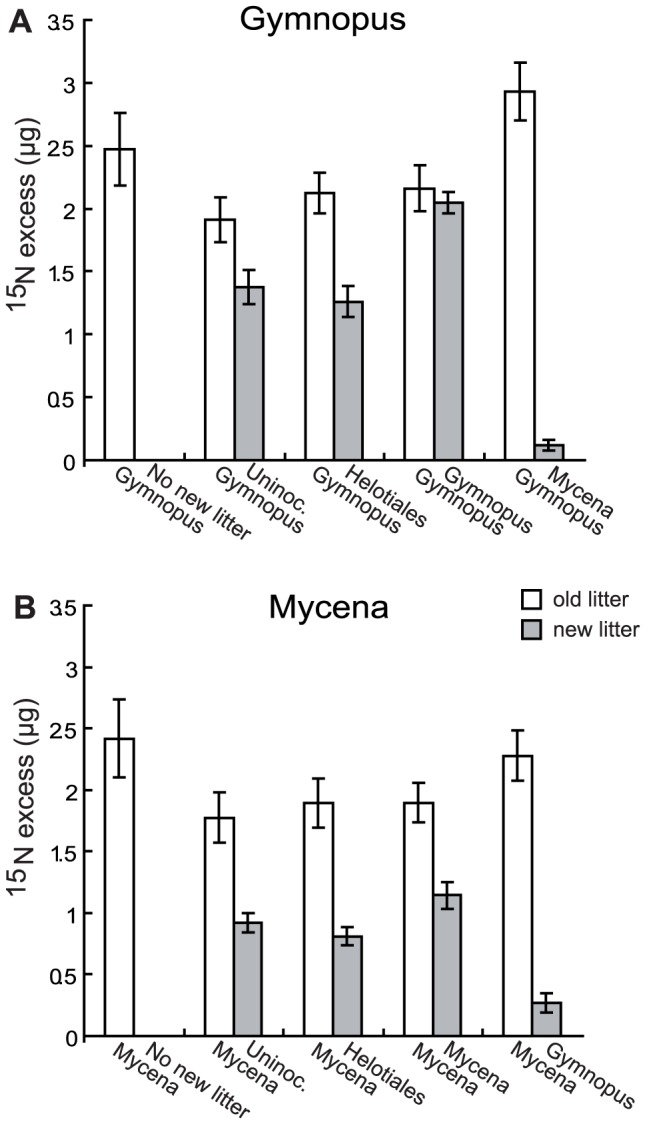
^15^N taken up and translocated by the fungi. ^15^N excess (μg in each litter bag) in litter bags containing needles colonized by litter degrading fungi. Open bars represent old litter incubated for a total of 10 months and grey bars represent new litter which was placed on top of the old litter after 5 months. The old litter was inoculated with (A) *Gymnopus androsaceus* or (B) *Mycena epipterygia*. The new litter was uninoculated or subjected to single-species or two-species interaction, i.e. the new litter was pre-inoculated with the same species, the other basidiomycete or a Helotiales sp. Data are mean ± SEM (for numbers of replicates, see Fig. 2).

**Figure 4:**
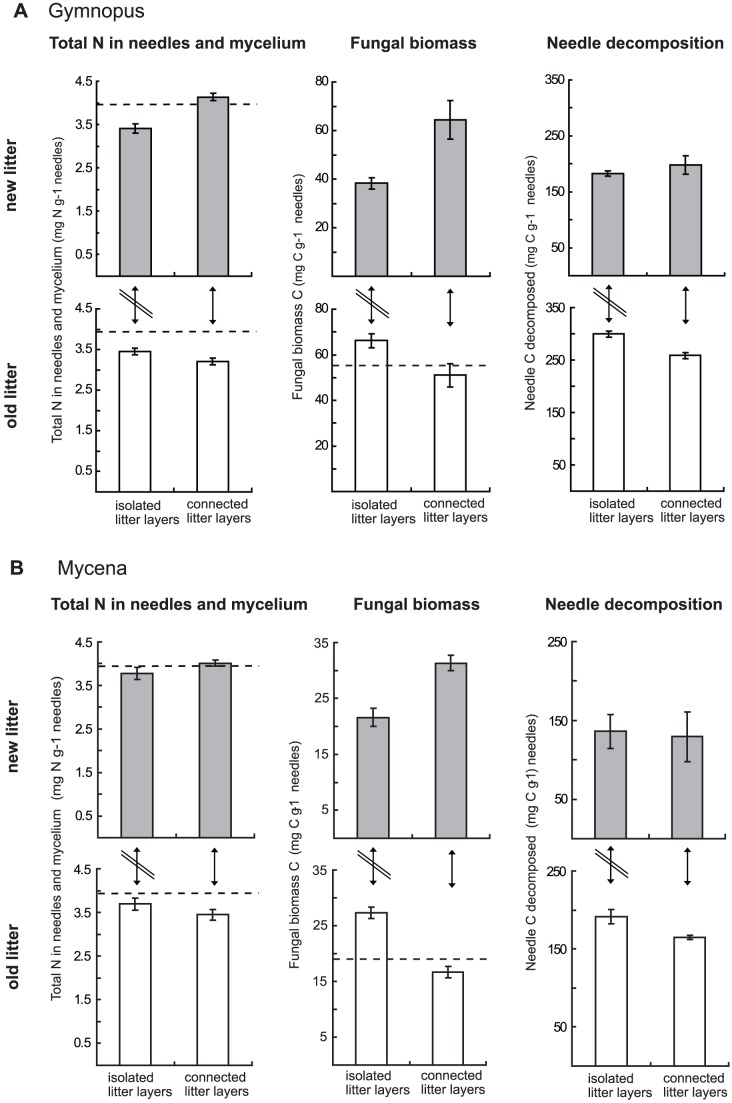
N-reallocation, mycelial growth and litter decomposition. Total N in needles and mycelium, total fungal biomass C produced and decomposed needle C (mass loss + mycelial C) of old litter (open bars) and new litter (grey bars) colonized by the litter degrading fungi *Gymnopus androsaceus* (A) or *Mycena epipterygia* (B). All data is given per g needles at the start of the experiment. ‘Isolated litter layers’ represents *old litter* and *new litter* incubated separately. In ‘connected litter layers’ the new litter was placed on top of the old litter after 5 months and then incubated together for an additional 5 months. Data are means ± SEM (for numbers of replicates, see Fig. 2) of systems receiving N during the first incubation period (data for systems without N addition is given in Table S1). In diagrams showing total N in needles and mycelium, the horizontal dashed line represents initial needle N content. In diagrams showing fungal biomass, the horizontal dashed line represents fungal biomass produced during the first 5 months of incubation.

ANOVA indicated that addition of NH_4_Cl to the sand during the first incubation period increased the average N content of the litter bags by 10% (*P* = 0.003; isolated bags only), leading to 18% higher mycelial production (*P* = 0.02) and 6% higher decomposition rates (*P* = 0.03; Table S1). At the end of the experiment, 20% of the added ^15^N was recovered in the isolated litter bags. Connected systems had taken up 22 – 27% of the added ^15^N, of which approximately one third had been reallocated to the upper, new litter (Fig. 3). Overall, litter inoculated with Gymnopus was more decomposed than litter inoculated with Mycena, and Gymnopus also produced more fungal mycelium (Fig. 4).

ANOVA indicated that when litters of different stages of decomposition were incubated together, the total amount of N in the upper, new litter, increased compared to litter incubated in isolation, but a significant connection x species interaction term followed by a Fisher's LSD test indicated that the difference was significant only for systems with Gymnopus (*P*<0.0001). Correspondingly, the amount of N in the lower, well decomposed litter decreased (*P* = 0.04; added N only due to missing data; Fig. 4a). In connected systems, higher fungal biomass was found in the new litter (*P*<0.0001) but lower biomass in the old litter (ANOVA followed by Fisher's LSD test: Gymnopus *P*<0.004; Mycena *P*<0.0001). In fact, in connected systems, fungal biomass in the old litter did not increase further during the second incubation period, whereas in isolated systems the fungi continued to grow also in the old litter (Fig. 4). In parallel, decomposition of the old litter decreased in response to litter addition (ANOVA followed by Fisher's LSD test: Gymnopus *P*<0.0001; Mycena *P* = 0.04), whereas there was no significant effect on decomposition of the new litter (Fig. 4).

### Effects of interspecific interactions

According to visual inspection of the systems at harvest, interspecific interactions (two-species) between the two basidiomycetes ended in deadlock, *i.e.* neither of the strains was able to enter the territory colonized by the other. In two-species basidiomycete interaction systems, only small amounts of added ^15^N were recovered from the new litter (Fig. 3), whereas in systems where the new litter was pre-inoculated with the same strain as the old litter (single-species), ^15^N reallocation to new litter was even greater than when non-inoculated litter was colonised from below.

In most respects, single-species systems with old and new litter behaved as systems where non-inoculated, new litter was colonised from below, whereas two-species basidiomycete interaction systems behaved as litters incubated in isolation (Table 1). ANOVA indicated that the amounts of N (*P* = 0.0008) and fungal biomass (*P* = 0.001) in the new litter were higher in single-species systems compared to two-species systems. In contrast, the amounts of N (*P* = 0.03), fungal biomass (significant only for systems with Mycena; Fisher's LSD *P* = 0.0005) and decomposition (*P* = 0.0002) in the old litter were lower in single-species systems compared to two-species systems. Mycena increased decomposition of new litter in two-species systems (*P* = 0.02; Table 1). In all measured aspects, the bags pre-colonized by the Helotialean strain behaved like bags where the basidiomycetes grew up into uncolonized new litter (c.f. Table 1 and Table S1).

**Table 1:** Single-species and two-species interactions.

	Old litter				New litter			
Gymnopus	Fungus	Total N (mg N g^−1^ needles)	Fungal biomass (mg C g^−1^ needles)	Needle decomposition (mg C g^−1^ needles)	Fungus	Total N (mg N g^−1^ needles)	Fungal biomass (mg C g^−1^ needles)	Needle decomposition (mg C g^−1^ needles)
Single-species interaction	Gymnopus	3.2±0.1	54.5±2.8	290.3±7.5	Gymnopus	4.1±0.1	71.4±6.4	235.5±11.4
Two-species ascomycete interaction	Gymnopus	3.5±0.2	58.8±7.9	268.2±6.1	Helotiales	4.1±0.1	69.5±4.8	216.1±3.0
Two-species basidiomycete interaction	Gymnopus	3.5±0.1	67.1±6.3	323.9±6.0	Mycena	3.5±0.1	49.0±5.3	217.5±10.4
**Mycena**								
Single-species interaction	Mycena	3.6±0.1	16.5±2.2	174.7±2.7	Mycena	4.2±0.1	28.4±2.4	131.4±6.1
Two-species ascomycete interaction	Mycena	3.7±0.2	20.2±3.5	171.4±4.5	Helotiales	4.1±0.1	30.1±3.3	118.7±5.9
Two-species basidiomycete interaction	Mycena	3.7±0.1	30.9±3.1	205.1±8.4	Gymnopus	3.6±0.3	20.5±1.4	163.8±6.2

Total N in needles and mycelium (Total N), total fungal biomass C produced and needle C decomposed (mass loss + mycelial C) of old litter and new litter, inoculated with *Gymnopus androsaceus*, *Mycena epipterygia* or Helotiales (new litter only). Old and new litters were combined after inoculation with the same (single-species) or different (two-species) fungal species. All data is given per g of needles at the start of the experiment. ‘Old litter’ represents needles incubated for a total of 10 month and ‘New litter’ represents needles which were placed on top of the old litter after 5 months. Data are mean ± SEM (for numbers of replicates, see Fig. 2).

### Losses of C and N to sand solution

**Figure 5:**
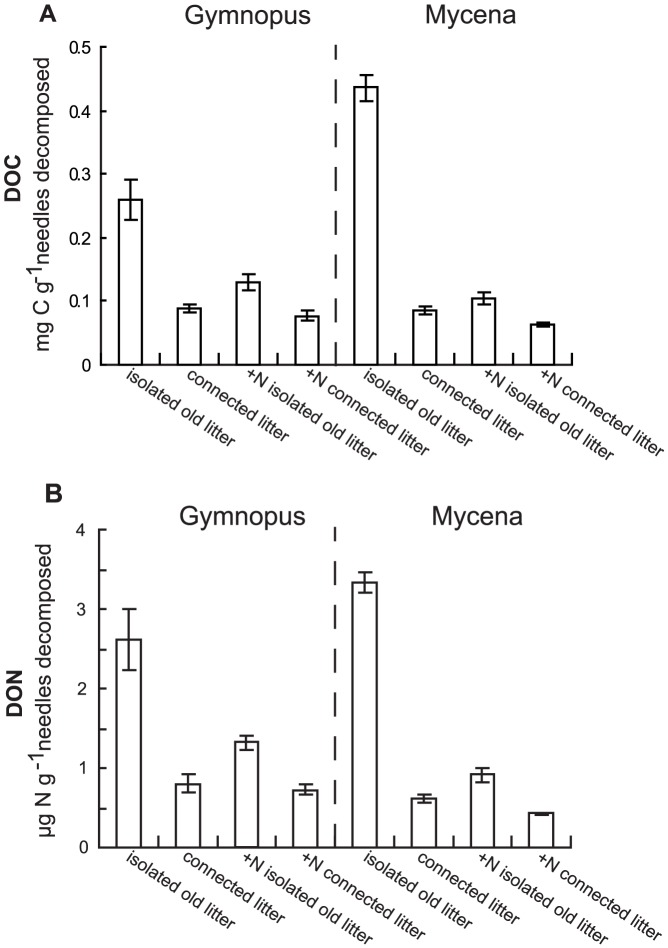
Dissolved organic C and N produced. Dissolved organic C (A) and dissolved organic nitrogen (B) in the sand solution of systems containing needles colonized by litter degrading fungi, related to the amounts of needle C degraded (mass loss + fungal C) during the last 5 months of a total 10 month incubation period. The systems were inoculated with either *Gymnopus androsaceus* or *Mycena epipterygia*. ‘Isolated systems’ represents systems with single litter bags incubated for 2×5 months (without and with N addition) and ‘connected litter’ represents systems with old litter receiving new litter during the second incubation period (without and with N addition during the first 5 month). Data are means ± SEM (for numbers of replicates, see Fig. 2).

Approximately 6% and 2% of the total N pool at harvest was recovered as DON in the sand solution of isolated litter bag systems inoculated with Gymnopus and Mycena, respectively. ANOVA indicated that there was a general negative effect of addition of new litter (*P*<0.001) on the amount of DOC and DON produced per g decomposed needle C (Fig. 5). In systems without added N, addition of new litter even decreased the *absolute* amounts of DOC and DON produced by the system, in spite of the larger amount of decomposed organic material (data not shown). N-addition during the first incubation period also had a negative effect on mg DOC and DON produced (significantly so in isolated litter bag systems only; Fisher's LSD *P*<0.001). Mixing model calculations based on the ^15^N content of the sand solution indicated that 60% and 33% of the DON originated from the fungi in systems inoculated with Gymnopus and Mycena, respectively. DON correlated significantly with DOC, with C:N ratios of 92 and 129 for the dissolved organic matter (DOM) produced in systems inoculated with Gymnopus and Mycena, respectively.

**Figure 6:**
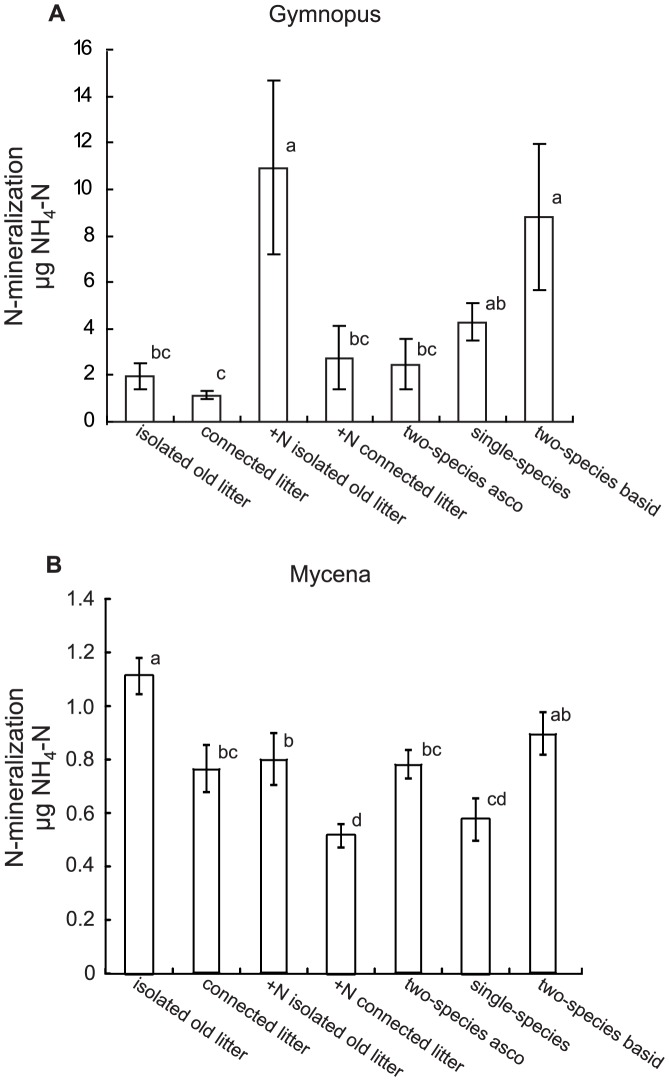
N mineralized from the pine needles. N-mineralization as amounts of NH_4_-N, produced during the last 5 months of a total 10 month incubation period, in the sand solution of systems containing needles colonized by litter degrading fungi *Gymnopus androsaceus* (A) and *Mycena epipterygia* (B). ‘Isolated systems’ represents systems with single litter bags incubated for 2×5 months (without and with N addition) and ‘connected litter’ represents systems with old litter receiving new litter during the second incubation period. ‘two-species asco’ represents systems where the new litter was pre-inoculated with Helotiales, ‘single-species’ represents systems where the new litter was pre-inoculated with the same species as the old litter and ‘two-species basid’ represents systems were the new litter was pre-inoculated with the other basidiomycete. Note the different scales on the y-axis in the two graphs. Data are mean ± SEM (for numbers of replicates, see Fig. 2). Bars sharing the same letters are not significantly different (P>0.05).

NH_4_ production in systems inoculated with Mycena was very low, equivalent to 0.07–0.6‰ of the total N and to 1–2% of the total dissolved N. N-mineralization by Gymnopus was variable, between treatments as well as among replicates, contributing 1 to 15% of the total dissolved N. The highest amounts of NH_4_ were found in the isolated litter bag systems and in the dead-lock interaction systems (Fig. 6).

### C-use efficiency

**Figure 7:**
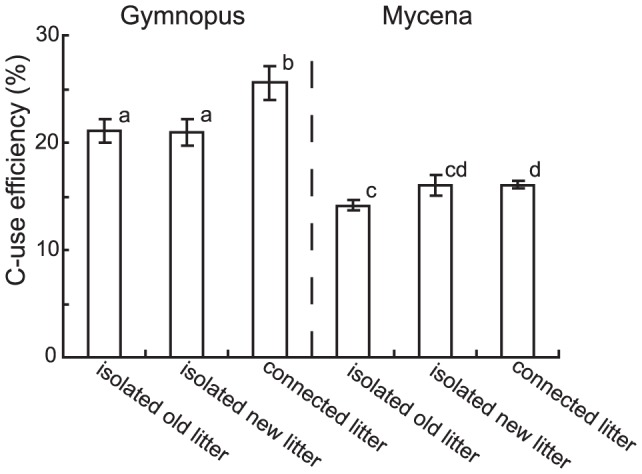
Fungal C-use efficiency. C-use efficiency calculated as mycelial C divided by the decomposed needle C (mass loss + mycelial C) at harvest for systems inoculated with *Gymnopus androsaceus* and *Mycena epipterygia*. ‘Isolated old litter’ represents needles incubated for 2×5 months, ‘isolated new litter’ represents needles incubated for 5 months and ‘connected litter’ represents double litter systems were old and new litter where incubated together and interconnected by a common mycelium. Data are mean ± SEM (for numbers of replicates, see Fig. 2). Bars sharing the same letters are not significantly different (*P*>0.05).

The average C-use efficiency (*i.e.* proportion of assimilated C allocated to biomass production) of Gymnopus across all systems was 25%, whereas the corresponding figure for Mycena was 16%. Gymnopus colonizing old and new litter had a higher C-use efficiency than the fungus colonizing old and new litter separately (Fig. 7). For Mycena, the C-use efficiency in isolated old litter was significantly lower than in connected litter. N addition had no significant effect on C-use efficiency (data not shown). The estimated C-use efficiencies should be regarded as a lower bound approximation, since mycelium in the sand was not accounted for.

### Establishment of C, N and chitin ratios

**Figure 8:**
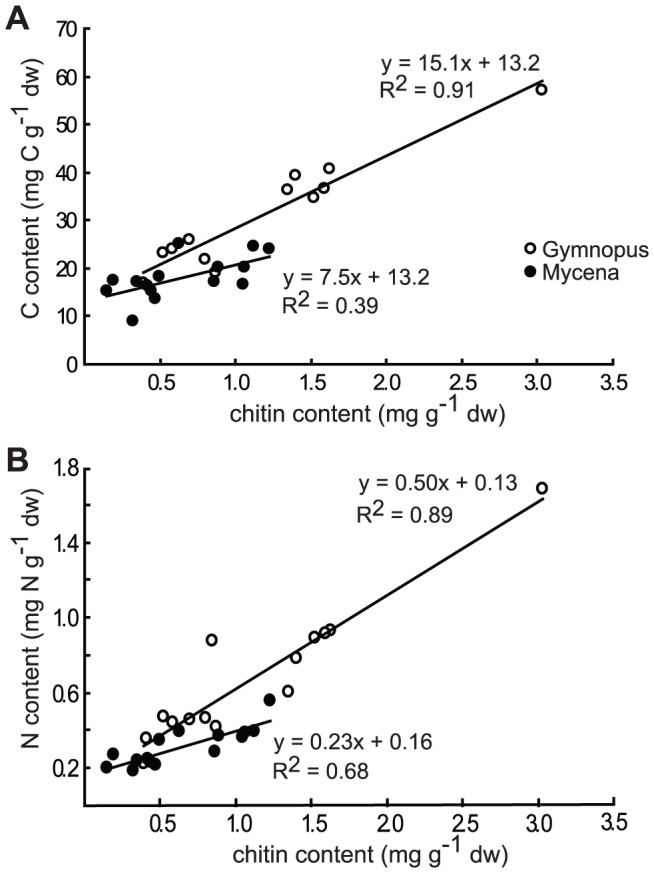
Chitin to C and N conversion. Relationship between (A) C content and chitin content and (B) N content and chitin content of glass fibre bundles retrieved from needle litter bags colonized by mycelium of *Gymnopus androsaceus* (open circles) and *Mycena epipterygia* (filled circles). Each data point represents pooled samples from five to eight system samples.

The amounts of chitin extracted from colonised glass wool bundles were linearly correlated to both the C and N content (Fig. 8). The intercepts of the C and N axes were interpreted as representing background levels of C and N. The relationships between chitin and fungal C in the glass wool bundles were consistent between treatments but differed between the two-species. The regression equations yielded a chitin to C conversion factor of 15.1 (r^2^ = 0.91) and a chitin to N conversion factor of 0.5 (r^2^ = 0.89) for Gymnopus. The chitin to C and chitin to N conversion factors for Mycena were estimated to 7.5 (r^2^ = 0.39) and 0.23 (r^2^ = 0.68), respectively. Consequently the C:N ratios of the fungal mycelium were estimated to 30 and 32 for Gymnopus and Mycena, respectively. ANOVA found no significant effects on the C:N ratio of the mycelium due to addition of N (*P* = 0.2) or incubation time (*P* = 0.9). Assuming a fungal C content of 50%, the fungal mycelium (dw) chitin contents were estimated to 3.3% for Gymnopus and 6.7% for Mycena.

## Discussion

### C and N dynamics and interaction between old and new litter

The results from the experiments supported our original hypothesis (i) that N would be reallocated from well decomposed needles to fresh litter (although the net increase in new litter was significant only for *Gymnopus*) and that increased N availability would stimulate fungal growth in the new litter. The observed stimulation of decomposer fungi by N addition (Table S1) is consistent with earlier observations of a stimulatory effect of N amendments on fungal growth and decomposition rates in laboratory studies [7], [8], [9] and supports that fungal growth and activity in coniferous litter is N limited at early stages of decomposition. Rapidly growing mycelium in recently deposited litter is likely to be a relatively strong sink for N that causes withdrawal of N from the older litter, suggesting internal competition for N between different parts of the mycelium [4]. The limited amount of ^15^N found in new litter of the interspecific interaction systems ending in deadlock indicates that the N redistribution was due to active mycelial transport rather than passive diffusion.

These results support earlier findings of fungal N import into decomposing litter [13], [15], which may substantially contribute to the net increases in N that are frequently detected in decomposing litter during early stages of decay [2], [10], [11], [12], [28]. During the second or third year of decomposition, the total N pool generally decreases [11], [29], [30], something that has often been interpreted as release of N to the soil solution. Instead, this decrease may be accounted for by reallocation of N from the old litter to support mycelial colonization of new substrates, in concordance with the lower N pool of old litter in our connected systems (Fig. 4). However, we found no support for increased decomposition of the new litter in response to this redistribution of N. This indicates that the increased fungal growth stemmed from more efficient C utilization (Fig. 7) rather than increased C acquisition, further highlighting the N-limitation of the fungi.

We further hypothesised (ii) that C would be reallocated from fresh litter to well degraded litter, increasing fungal growth and needle decomposition in the old litter. We did not determine C translocation explicitly, but stagnating fungal growth in the old litter after addition of new litter, indirectly refutes our hypothesis. In fact, biomass in the old litter even appeared to decrease, most likely due to autolysis and enzymatic self-degradation in order to recycle N [31]. Thus, rather than supporting mycelial growth in old litter, addition of new litter led to redistribution of fungal biomass and N (Fig. 4) and reduced decomposition of the old litter. Similarly, decay rates of wood blocks by wood decomposing fungi have been found to decrease in response to colonization of fresh substrates [32].

This redistribution of fungal growth most likely occurred in response to the higher relative quality of the new litter. Old litter colonized by Gymnopus approached 35% loss of needle C after 5 months of incubation in the laboratory, which corresponds to mass loss observed after approximately 1.3 years in the field [2]. Nevertheless, isolated litter bags could support additional fungal growth during the second incubation period. Thus, the fungi were still capable of utilizing the old litter, but reallocated their growth and activity to newly added litter when this was available.

The observation that N-limited decomposer fungi may leave residual but potentially usable C has important implications for our understanding of C sequestration in forest ecosystems and may contribute to the discussion why not all C is degraded in soils [33], [34]. The concept of absolute recalcitrance of organic matter, *i.e.* that C accumulates in ecosystems because it is resistant to decomposition, has recently been challenged by Schmidt *et al*. [35], who instead emphasise environmental and biological control of organic matter accumulation. Accumulation of old C when more attractive C-sources are available could be described in terms of a ‘negative priming effect’ [36] and could potentially contribute to organic matter accumulation in the litter layers of boreal forests.

We found that the C-use efficiency of Gymnopus increased due to addition of new litter (Fig. 7), but this was not directly related to the better quality of the new litter, since the efficiency did not differ significantly between isolated litter-bags incubated for one or two periods. Rather, the higher C-use efficiency may be attributed to resource reallocation. If degradation is driven by N demand, import of additional N into fresh litter could stimulate fungal growth without a concomitant increase in degradation, as here observed. Without extra N, C is available in excess of demand and is removed by respiration, leading to lower C-use efficiency [7]. Thus, redistribution of N may lead to better balanced C and N availability and a more efficient use of mobilised C. In fact, in systems inoculated with Gymnopus, mycelial connection reduced overall needle decomposition by 6% but increased fungal biomass by up to 19%. This highlights the fact that decomposer fungi have not evolved to maximize decomposition rates. Rather, maximizing growth and the potential to explore new substrates and ultimately produce fruit bodies may be the primary capability that promotes evolutionary success.

### Losses of dissolved N

The observation that small amounts of N were lost to the sand solution in the studied systems, with a major fraction lost as high C:N ratio dissolved organic matter and only minute amounts as NH_4_, is in agreement with the patterns of high N immobilisation rates generally observed in ecosystems of low productivity [37], [38]. The pool of total dissolved N in the sand could, however, not account for total N losses from the needle-mycelium complex. Presumably, some additional N may be attributed to fungal growth and particulate matter in the sand. Additions of N during the first incubation period decreased the relative losses of soluble organic matter per unit degraded C during the second period (Fig. 5). In fact, the absolute amounts of DON released during the second incubation period were lower in systems that had received N during the first period. Addition of new litter also had a negative effect on soluble losses, suggesting that stimulation of fungal growth restricted N losses and lead to more efficient resource utilization.

In Gymnopus, relatively high N-mineralization rates were found in isolated litter bag systems and in the basidiomycete species interaction systems compared to connected systems and single-species interaction systems. Thus, in un-connected systems where N could not be translocated away from old litter, C-limitation resulted in N-mineralization, but in connected systems, mineralization was minimal in agreement with our original hypothesis (ii). Using agar cultures, we have previously showed that C translocation between spatially separated substrates may drastically reduce N-mineralization in mycelium growing on low C:N ratio medium [9], [39]. Here, we corroborate that mycelial translocation may restrict N-mineralization also in more realistic systems with natural substrates of different qualities.

In undisturbed boreal forest ecosystems, coniferous litter decomposes over a long time period, maintaining a continuing supply of C. Consequently, boreal litter decomposing fungi are not likely to release large amounts of inorganic nutrients for plant uptake [38], [40], contradicting the normally perceived role of saprotrophic microorganisms in N cycling. Instead, litter fungi appear to retain assimilated N in order to maximize biomass production in the upper litter layer. Mycelial N is also continuously lost to grazing soil fauna [41]. Residual N that the litter decomposers fail to reallocate presumably enters the humus pool in the form of fungal cell wall compounds stabilized in complex organic forms [42], [43], [44]. N mobilisation from well degraded organic matter then has to rely on mycorrhizal fungi to support plant production from organic sources without previous mineralization [40], [45].

### Effects of interspecific interactions

In the present study, interaction between the two basidiomycetes seemed to have prevented colonization of new litter by the fungus from the old litter (Fig. 3, Table 1). These systems, thereby, served as a ‘non-translocation control’ with similar responses as in isolated litter bags, confirming our original hypothesis (iii). Territorial behaviour and strong antagonism has been well described for wood decomposing fungi [19]. Indirect evidence of antagonism between litter degrading fungi has previously been presented by Newell, [46], who found that *Mycena galopus* expanded its spatial niche upwards when the more surficial *Gymnopus* was suppressed by selective grazing. Here we confirm that strong antagonism may take place between interacting litter basidiomycetes, with major negative effects on connectivity between resources. However, dead-lock may not be a general outcome of interactions; with other interacting species or a different balance in relative resource availability, interactions may likely result in over-growth of one species by another [19], [47], with a different outcome in terms of nutrient reallocation.

The presence of the ascomycete in new litter did not alter the behaviour of the basidiomycetes in any way (Table 1), and it appeared to have a low competitive strength. Similar to many other litter colonising ascomycetes, the Helotiales strain has previously showed limited degradation of cellulose and lignin [23]. The ecological strategies by which helotialean ascomycetes co-exist in needles with highly territorial and more efficient basidiomycetes remain unclear.

High small scale diversity in fungal communities and the resulting multitude of interactions may act to alter nutrient reallocation, and when extrapolating to the field situation, the results of this laboratory study have to be considered in the context of fungal succession and community dynamics [18], [48].

### C and N content of fungal mycelium

The chitin to C conversion factors of 15.1 and 7.5 (Fig. 8) and fungal mycelial chitin contents of 3.3 and 6.7% for Gymnopus and Mycena, respectively, fall within the same range as earlier estimates [49], [50]. The estimated C:N ratios of 30 and 32 for the mycelium of Gymnopus and Mycena, respectively, are much higher than the ratio of 10–15 generally stated for soil fungi [51]. Nevertheless, Boddy & Watkinson [52] report a C:N ratio of 35 for a typical wood decomposing fungus, and the C:N ratio of mycorrhizal fungi has been estimated to be 20 [53]. In addition, the N-content of fungal mycelium may show a high degree of plasticity depending on environmental conditions [54], [55].

In the glass wool bundles, the correlations between chitin and both C and N in mycelium of Gymnopus were strong (Fig. 7; r^2^ = 0.91 and r^2^ = 0.89). Estimated using the chitin to N factor, fungal biomass N constituted between 32% of the total N pool in isolated bags without N after 5 months incubation and 55% in old litter of connected systems after 10 months incubation. Together with the high proportion of DON in the sand solution derived from mycelium (60%), these data indicate that a large fraction of the N in the needles had been assimilated by the fungus at the end of the experiment, again highlighting the N-limitation of the fungi.

The C-use efficiencies estimated for Gymnopus and Mycena in these microcosm systems were 25% and 16%, respectively. These estimates depend heavily on the chitin to C conversion factors and should, thus, be regarded as rough estimates. The estimated C-use efficiencies are comparable to the efficiency rates of 28–34% found in *Mycena galopus* decomposing deciduous litter [50].

## Conclusions

We here show that the capacity of litter decomposing fungi to translocate resources can have a major influence on storage and release of C and N in interacting litters. When different litter C pools interacted through fungal connections, older more decomposed litter was left behind due to a relative difference in quality rather than due to absolute recalcitrance. Such preferential decomposition of high quality C may take place when decomposer fungi experience over-all N-limitation. From an ecosystem point of view, this phenomenon could potentially contribute to organic matter accumulation in the litter layers of boreal forests.

N mineralization and DON release was minimized in the presence of fresh litter, suggesting that fungal litter degraders primarily act to redistribute rather than release N in boreal forests. This reductionistic laboratory study highlights properties of decomposer systems dominated by translocating fungi that, if properly represented, may improve the predictive power of quantitative ecosystem models. Our results raise new hypotheses that call for further exploration under more realistic field conditions.

## Supporting Information

Table S1 **N-reallocation, mycelial growth and litter decomposition in systems without and with added N.** Total N in needles and mycelium, total fungal biomass C produced and needle C decomposed (mass loss + fungal C) of old litter (incubated for 2×5 months) and new litter (incubated for 5 months), inoculated with litter degrading fungi Gymnopus androsaceus (Gymnopus) or Mycena epipterygia (Mycena). All data is given per g of needles at the start of the experiment. In ‘New litter’, ‘isolated’ represents isolated litter bags incubated on sand during the second incubation period only and ‘on litter’ represents new litter placed on top of the corresponding old litter (with or without N addition). In ‘old litter’, isolated represents isolated litter bags incubated for two periods (with and without N addition) and ‘+ new litter’ represents old litter receiving new litter during the second incubation period. Data are means ± SEM (for numbers of replicates, see Fig. 1).
(DOC)

## References

[1] Lindahl BD, Ihrmark K, Boberg J, Trumbore SE, Högberg P, et al (2007) Spatial separation of litter decomposition and mycorrhizal nitrogen uptake in a boreal forest. New Phytologist 173: 611–620. 17244056 10.1111/j.1469-8137.2006.01936.x

[2] Berg B, Hannus K, Popoff T, Theander O (1982) Changes in organic chemical components of needle litter during decomposition. Long-term decomposition in a Scots pine forest. I. Canadian Journal of Botany 60: 1310–1319.

[3] Boddy L (1999) Saprotrophic cord-forming fungi: meeting the challenge of heterogeneous environments. Mycologia 91: 13–32.

[4] Lindahl BD, Olsson S (2004) Fungal translocation-creating and responding to environmental heterogeneity. Mycologist 18: 79–88.

[5] Tamm CO (1991) Nitrogen in Terrestrial Ecosystems-questions of productivity, vegetational changes, and ecosystem stability; Billings WD, Golley F, Lange OL, Olson JS, Remmert H, editors. Berlin: Springer-Verlag.

[6] Berg B (2000) Litter decomposition and organic matter turnover in northern forest soils. Forest ecology and Management 133: 13–22.

[7] Boberg J, Finlay RD, Stenlid J, Nasholm T, Lindahl BD (2008) Glucose and ammonium additions affect needle decomposition and carbon allocation by the litter degrading fungus *Mycena epipterygia* . Soil Biology & Biochemistry 40: 995–999.

[8] Allison SD, LeBauer DS, Ofrecio MR, Reyes R, Ta A-M, et al (2009) Low levels of nitrogen addition stimulate decomposition by boreal forest fungi. Soil Biology & Biochemistry 41: 293–302.

[9] Boberg JB, Nasholm T, Finlay RD, Stenlid J, Lindahl BD (2011) Nitrogen availability affects saprotrophic basidiomycetes decomposing pine needles in a long term laboratory study. Fungal Ecology 4: 408–416.

[10] Fahey TL, Yavitt JB, Pearson JA, Knight DH (1985) The nitrogen cycle in lodgepole pine forests, souteastern Wyoming. Biogeochemistry 1: 257–275.

[11] Melillo JM, Aber JD, Linkins AE, Ricca A, Fry B, et al (1989) Carbon and nitrogen dynamics along the decay continuum - plant litter to soil organic-matter. Plant and Soil 115: 189–198.

[12] Moore TR, Trofymow JA, Prescott CE, Fyles J, Titus BD (2006) Patterns of carbon, nitrogen and phosphorus dynamics in decomposing foliar litter in Canadian forests. Ecosystems 9: 46–62.

[13] Frey SD, Elliott ET, Paustian K, Peterson GA (2000) Fungal translocation as a mechanism for soil nitrogen inputs to surface residue decomposition in a no-tillage agroecosystem. Soil Biology & Biochemistry 32: 689–698.

[14] Hart SC, Firestone MK (1990) Forest floor- mineral soil interactions in the internal nitrogen cycle of an old-growth forest. Biogeochemistry 12: 73–97.

[15] Zeller B, Colin-Belgrand M, Dambrine E, Martin F (1998) ^15^N-partitioning and production of ^15^N-labelled litter in beech trees following [^15^N]urea spray. Annales Des Sciences Forestieres 55: 375–383.

[16] Lindahl B, Boberg J (2008) Distribution and function of litter basidiomycetes in coniferous forests. In: Boddy L, Frankland JC, Van West P, editors. Ecology of Saprotrophic Basidiomycetes. London: Elsevier Ltd. pp. 183–196.

[17] Boberg J (2009) Litter decomposing fungi in boreal forests-their function in carbon and nitrogen circulation. Uppsala: Swedish University of Agricultural Sciences. 67 p.

[18] Baldrian P, Kolarik M, Stursova M, Kopecky J, Valaskova V, et al (2012) Active and total microbial communities in forest soil are largely different and highly stratified during decomposition. ISME journal 6: 248–258. 21776033 10.1038/ismej.2011.95 PMC3260513

[19] Boddy L (2000) Interspecific combative interactions between wood-decaying basidiomycetes. Fems Microbiology Ecology 31: 185–194. 10719199 10.1111/j.1574-6941.2000.tb00683.x

[20] Woodward S, Boddy L (2008) Interactions between saprotrophic fungi. In: Boddy L, Frankland JC, Van West P, editors. Ecology of Saprotrophic Basidiomycetes. London: Elsevier Ltd. pp. 125–141.

[21] Frankland JC (1998) Fungal succession-unravelling the unpredictable. Mycological Research 102: 1–15.

[22] O'Brien HE, Parrent JL, Jackson JA, Moncalvo JM, Vilgalys R (2005) Fungal community analysis by large-scale sequencing of environmental samples. Applied and Environmental Microbiology 71: 5544–5550. 16151147 10.1128/AEM.71.9.5544-5550.2005 PMC1214672

[23] Boberg JB, Ihrmark K, Lindahl BD (2011) Decomposing capacity of fungi commonly detected in *Pinus sylvestris* needle litter. Fungal Ecology 4: 110–114.

[24] Axelsson B, Bråkenhielm S (1980) Investigation sites of the Swedish coniferous forest project- biological and physiograpical features. Ecological Bulletins 32: 25–64.

[25] Johansson M-B, Berg B, Meentemeyer V (1995) Litter mass-loss rates in late stages of decomposition in a climate transect of pine forest. Long-term decomposition in a Scots pine forest. IX. Can J Bot 73: 1509–1521.

[26] Ekblad A, Näsholm T (1996) Determination of chitin in fungi and mycorrhizal roots by an improved HPLC analysis of glucosamine. Plant and Soil 178: 29–35.

[27] Fry B (2006) Stable Isotope Ecology. New York: Springer. 308 p.

[28] Chadwick DR, Ineson P, Woods C, Piearce TG (1998) Decomposition of *Pinus sylvestris* litter in litter bags: Influence of underlying native litter layer. Soil Biology & Biochemistry 30: 47–55.

[29] Berg B, Staaf H (1981) Leaching, Accumulation and Release of Nitrogen in Decomposing Forest Litter. In: Clark FE, Rosswall T, editors. Terrestrial Nitrogen Cycles. Stockholm: Swedish Natural Science research Council. pp. 163–178.

[30] Gebauer G, Zeller B, Schimdt G, Buchmann N, Colin-Belgrand M, et al. (2000) The fate of ^15^N-labelled nitrogen inputs to coniferous and broadleaf forests. In: Schulze E-D, editor. Carbon and nitrogen cycling in european forest ecosystems. Heidelberg: Springer Verlag. pp. 144–170.

[31] Lindahl BD, Finlay RD (2006) Activities of chitinolytic enzymes during primary and secondary colonization of wood by basidiomycetous fungi. New Phytologist 169: 389–397. 16411941 10.1111/j.1469-8137.2005.01581.x

[32] Dowson CG, Springham P, Rayner ADM, Boddy L (1989) Resource relationships of foraging mycelial systems of *Phanerochaete velutina* and *Hypholoma fasciculare* in soil New Phytologist. 111: 501–509. 10.1111/j.1469-8137.1989.tb00713.x 33874011

[33] Ekschmitt K, Liu M, Vetter S, Fox O, Wolters V (2005) Strategies used by soil biota to overcome soil organic matter stability - Why is dead organic matter left over in the soil? Geoderma 128: 167–176.

[34] Allison SD (2006) Brown ground: A soil carbon analogue for the green world hypothesis? American naturalist 167: 619–627. 10.1086/503443 16671007

[35] Schmidt MWI, Torn MS, Abiven S, Dittmar T, Guggenberger G, et al (2011) Persistence of soil organic matter as an ecosystem property. Nature 478: 49–56. 21979045 10.1038/nature10386

[36] Kuzyakov Y, Friedel JK, Stahr K (2000) Review of mechanisms and quantification of priming effects. Soil Biology & Biochemistry 32: 1485–1498.

[37] Northup RR, Yu ZS, Dahlgren RA, Vogt KA (1995) Polyphenol control of nitrogen release from pine litter. Nature 377: 227–229.

[38] Schimel JP, Bennet J (2004) Nitrogen mineralization: Challenges of a changing paradigm. Ecology 85: 591–602.

[39] Boberg JB, Finlay RD, Stenlid J, Lindahl BD (2010) Fungal C translocation restricts N-mineralization in heterogeneous environments. Functional Ecology 24: 454–459.

[40] Lindahl BO, Taylor AFS, Finlay RD (2002) Defining nutritional constrains on carbon cycling in boreal forests - towards a less ‘phytocentric’ perspective. Plant and soil 242: 123–135.

[41] Lenoir L, Persson T, Bengtsson J, Wallander H, Wiren A (2007) Bottom-up or top-down control in forest soil microcosms? Effects of soil fauna on fungal biomass and C/N mineralisation. Biology and Fertility of Soils 43: 281–294.

[42] Piccolo A, Nardi S, Concheri G (1996) Micelle-like conformation of humic substances as revealed by size exclusion chromatography. Chemosphere 33: 595–602. 8759306 10.1016/0045-6535(96)00210-x

[43] Simpson AJ, Kingery WL, Hayes MHB, Spraul M, Humpfer E, et al (2002) Molecular structures and associations of humic substances in the terrestrial environment. Naturwissenschaften 89: 84–88. 12046627 10.1007/s00114-001-0293-8

[44] Clemmensen KE, Bahr A, Ovaskainen O, Dahlberg A, Ekblad A, et al (2013) Roots and associated fungi drive long-term carbon sequestration in boreal forest. Science 339: 1615–1618. 23539604 10.1126/science.1231923

[45] Read DJ, Perez-Moreno J (2003) Mycorrhizas and nutrint cycling in ecosystems- a journey toward relevance. New Phytologist 157: 475–492. 10.1046/j.1469-8137.2003.00704.x 33873410

[46] Newell K (1984) Interaction between two decomposer basidiomycetes and a collembolan under sitka spruce -grazing and its potential effects on fungal distribution and litter decomposition Soil Biology & Biochemistry. 16: 235–239.

[47] Robinson CH, Dighton J, Frankland JC, Coward PA (1993) Nutrient and carbon dioxide release by interacting species of straw-decomposing fungi. Plant and Soil 151: 139–142.

[48] van der Wal A, Geydan TD, Kuyper TW, de Boer W (2013) A thready affair: linking fungal diversity and community dynamics to terrestrial decomposition processes. FEMS Microbiology Reviews 37: 477–494. 22978352 10.1111/1574-6976.12001

[49] Appuhn A, Joergensen RG (2006) Microbial colonisation of roots as a function of plant species. Soil Biology & Biochemistry 38: 1040–1051.

[50] Frankland JC, Lindley DK, Swift MJ (1978) A comparison of two methods for estimation of mycelial biomass in leaf litter Soil Biology & Biochemistry. 10: 323–333.

[51] Paul EA (2007) Soil microbiology, ecology and biochemistry. Canada: Academic Press. 532 p.

[52] Boddy L, Watkinson SC (1995) Wood decomposition, higher fungi, and their role in nutrient redistribution. Canadian Journal of Botany 73: S1377–S1383.

[53] Wallander H, Nilsson LO, Hagerberg D, Rosengren U (2003) Direct estimates of C:N ratios of ectomycorrhizal mycelia collected from Norway spruce forest soils. Soil Biology & Biochemistry 35: 997–999.

[54] Levi MP, Cowling EB (1969) Role of nitrogen in wood deterioration. VII. Physiological adaption of wood-destroying and other fungi to substrate deficient nitrogen. Phytopathology 59: 460–468.

[55] Venables CE, Watkinson SC (1989) Medium-induced changes in patterns of free and combined amino-acids in mycelium of *Serpula lacrymans* . Mycological Research 92: 273–277.

