# Supplementary material for: Nitrogen and Carbon Reallocation in Fungal Mycelia during Decomposition of Boreal Forest Litter
Source: PLoS One. 2014 Mar 20;9(3):e92897. doi: 10.1371/journal.pone.0092897 (PMC3961408; doi:10.1371/journal.pone.0092897)
Supplement: Table S1 — N-reallocation, mycelial growth and litter decomposition in systems without and with added N. Total N in needles and mycelium, total fungal biomass C produced and needle C decomposed (mass loss + fungal C) of old litter (incubated for 2×5 months) and new litter (incubated for 5 months), inoculated with litter degrading fungi Gymnopus androsaceus (Gymnopus) or Mycena epipterygia (Mycena). All data is given per g of needles at the start of the experiment. In ‘New litter’, ‘isolated’ represents isolated litter bags incubated on sand during the second incubation period only and ‘on litter’ represents new litter placed on top of the corresponding old litter (with or without N addition). In ‘old litter’, isolated represents isolated litter bags incubated for two periods (with and without N addition) and ‘+ new litter’ represents old litter receiving new litter during the second incubation period. Data are means ± SEM (for numbers of replicates, see Fig. 1). (DOC) [file pone.0092897.s001.doc]

***Table S1.*** ***N-reallocation, mycelial growth and litter decomposition in systems without and with added N.***

|  | **Gymnopus** | | | **Mycena** | | |
| --- | --- | --- | --- | --- | --- | --- |
| **Treatment** | **Total N in needles and mycelium (mg N g^-1^ needles)** | **Fungal biomass (mg C g^-1^ needles)** | **Needle decomposition (mg C g^-1^ needles)** | **Tot N in needles and mycelium (mg N g^-1^ needles)** | **Fungal biomass (mg C g^-1^ needles)** | **Needle decomposition (mg C g^-1^ needles)** |
| ***New litter*** |  |  |  |  |  |  |
| isolated layer | 3.4 ± 0.1 | 38.3 ± 2.4 | 182.7 ± 5.1 | 3.8 ± 0.1 | 21.6 ± 1.6 | 136.0 ± 6.5 |
| on old litter | 4.1 ± 0.1 | 62.1 ± 3.7 | 188.0 ± 7.4 | 4.0 ± 0.1 | 33.2 ± 3.1 | 131.3 ± 8.2 |
| on old litter (N) | 4.1 ± 0.1 | 64.3 ± 7.9 | 198.0 ± 16.4 | 4.0 ± 0.1 | 31.3 ± 1.4 | 129.2 ± 3.1 |
| ***Old litter*** |  |  |  |  |  |  |
| isolated layer | 2.9 ± 0.2 | 54.0 ± 5.7 | 279.5 ± 8.8 | 3.3 ± 0.1 | 23.7 ± 1.3 | 171.1 ± 2.5 |
| + new litter | nd | 48.0 ± 6.5 | 244.2 ± 4.7 | 3.2 ± 0.2 | 15.0 ± 0.9 | 173.6 ± 8.8 |
| isolated layer +N | 3.5 ± 0.1 | 66.2 ± 3.1 | 299.8 ± 5.6 | 3.7 ± 0.1 | 27.3 ± 1.0 | 191.5 ± 9.3 |
| + new litter +N | 3.2 ± 0.1 | 51.0 ± 5.0 | 258.4 ± 5.7 | 3.5 ± 0.1 | 16.7 ± 1.0 | 164.6 ± 2.9 |

Total N in needles and mycelium, total fungal biomass C produced and needle C decomposed (mass loss + fungal C) of old litter (incubated for 2 x 5 months) and new litter (incubated for 5 months), inoculated with litter degrading fungi *Gymnopus androsaceus* (Gymnopus) or *Mycena epipterygia* (Mycena). All data is given per g of needles at the start of the experiment. In ‘New litter’, ‘isolated’ represents isolated litter bags incubated on sand during the second incubation period only and ‘on litter’ represents new litter placed on top of the corresponding old litter (with or without N addition). In ‘old litter’, isolated represents isolated litter bags incubated for two periods (with and without N addition) and ‘+ new litter’ represents old litter receiving new litter during the second incubation period. Data are means ± SEM (for numbers of replicates, see Fig. 1).
